# Multidrug-Resistant Tuberculosis  Outbreak among US-bound Hmong  Refugees, Thailand, 2005

**DOI:** 10.3201/eid1411.071629

**Published:** 2008-11

**Authors:** John E. Oeltmann, Jay K. Varma, Luis Ortega, Yecai Liu, Thomas O’Rourke, Maria Cano, Theresa Harrington, Sean Toney, Warren Jones, Samart Karuchit, Lois Diem, Dhanida Rienthong, Jordan W. Tappero, Kashef Ijaz, Susan A. Maloney

**Affiliations:** Centers for Disease Control and Prevention, Atlanta, Georgia, USA (J.E. Oeltmann, L. Ortega, Y. Liu, M. Cano, T. Harrington, S. Toney, L. Diem, K. Ijaz, S.A. Maloney); Thailand Ministry of Public Health–US Centers for Disease Control and Prevention Collaboration, Bangkok, Thailand (J.K. Varma, S. Karuchit, J.W. Tappero); The International Organization for Migration, Bangkok (T. O’Rourke, W. Jones); Thailand Ministry of Public Health, Bangkok (D. Rienthong)

**Keywords:** Refugees, outbreak, multidrug-resistant tuberculosis, tuberculosis screening, research

## Abstract

In January 2005, tuberculosis (TB), including multidrug-resistant TB (MDR TB), was reported among Hmong refugees who were living in or had recently immigrated to the United States from a camp in Thailand. We investigated TB and drug resistance, enhanced TB screenings, and expanded treatment capacity in the camp. In February 2005, 272 patients with TB (24 MDR TB) remained in the camp. Among 17 MDR TB patients interviewed, 13 were found to be linked socially. Of 23 MDR TB isolates genotyped, 20 were similar according to 3 molecular typing methods. Before enhanced screening was implemented, 46 TB cases (6 MDR TB) were diagnosed in the United States among 9,455 resettled refugees. After enhanced screening had begun, only 4 TB cases (1 MDR TB), were found among 5,705 resettled refugees. An MDR TB outbreak among US-bound refugees led to importation of disease; enhanced pre-immigration TB screening and treatment decreased subsequent importation.

## Abstract 2

Enhanced pre-immigration screening and program expansion decreased TB importation.

Globally, 9 million new cases of tuberculosis (TB) were reported in 2004 (*1*), ≈4.3% of which were multidrug-resistant TB (MDR TB) (*2*). MDR TB, defined as infection with *Mycobacterium tuberculosis* resistant to at least isoniazid and rifampin, complicates TB control efforts because it requires prolonged treatment with drugs that are less potent, more costly, and more toxic than traditional isoniazid- and rifampin-based regimens (*3*,*4*). These factors challenge implementation of effective TB control programs, particularly in developing countries.

Currently, 56% of TB cases in the United States occur in foreign-born persons (*5*), and rates are highest among recently arrived immigrants (*6*,*7*). Refugee populations are particularly vulnerable to TB and drug-resistant TB (*8*–*12*). Annually, 50,000–70,000 refugees immigrate to the United States (*13*); before entry, they must undergo standard pre-immigration screening for TB. Despite screening, however, refugee populations have exhibited high TB incidence rates shortly after arrival in the United States (*14*–*16*). One contributor to high postarrival TB rates is the low sensitivity of the current pre-immigration TB screening algorithm, which has been estimated to identify <35% of all TB cases (*17*).

In December 2003, the US Department of State announced a refugee resettlement program for ≈16,000 Hmong refugees from Laos, who had been living in a temporary camp for displaced persons in Lopburi, Thailand, for >10 years. The first refugees arrived in the United States in June 2004; by January 2005, ≈10,000 had immigrated to the United States. Initial reports, after health assessments of newly arrived refugees, identified 37 TB cases, 4 of which were MDR TB (*18*). Simultaneously, cases of MDR TB were confirmed in Thailand in refugees awaiting resettlement. Immigration to the United States was temporarily halted in January 2005, while the US Centers for Disease Control and Prevention (CDC) and international partners investigated the factors that led to emergence and dissemination of TB, including MDR TB, among these refugees. Results from the investigation guided implementation of enhanced TB screening and treatment for the ≈6,000 refugees remaining in the camp in Thailand.

## Materials and Methods

This investigation was deemed an urgent public health response. Under the federal regulation for the protection of human research participants, Code of Federal Regulations Title 45, part 46, this investigation was determined by CDC to not be human subject research.

### Case Definition and Case Detection

Cases were defined by positive sputum smears or cultures or by a physician’s decision to initiate TB treatment in the context of radiographic abnormalities and clinical features consistent with TB. From April 2004 through January 2005, pre-immigration TB screening in the camp detected TB cases among the refugees. The TB screening algorithm used initially consisted of a medical history, a physical examination, and, for applicants >15 years of age, a chest radiograph. Persons whose clinical or radiographic findings suggested TB disease submitted 3 sputum specimens for acid-fast bacilli smear microscopy. Limited laboratory capacity was available, and mycobacterial culture was performed on sputum samples that were smear positive for acid-fast bacilli. Refugees with smear-positive results were allowed to travel to the United States after they had begun anti-TB treatment and had smear-negative results for 3 follow-up sputum specimens. In July 2004, after a site visit by CDC and because of concerns about potential high prevalence of TB, including drug-resistant TB, the pre-immigration screening algorithm was expanded to include mycobacterial culture and drug-susceptibility testing for both smear-negative and smear-positive specimens. From February 2005 (after the TB outbreak was detected) through April 2007, suspected TB cases were also identified through contact tracing.

### Patient Interviews

Hmong interpreters interviewed TB patients in the camp, using a 24-item questionnaire about history of previous TB diagnosis and treatment. When asked about previous TB treatment, participants were shown anti-TB medications and asked if they had ever taken any of the displayed pills in the past. Patient responses were stratified by drug-susceptibility testing results. To assess the possibility of recent MDR TB transmission in the camp, MDR TB patients were asked additional questions regarding social links to other known MDR TB patients. A strong link was defined as sharing a household or having contact with another MDR TB patient at least 1×/week. A weak link was defined as having contact with another MDR TB patient <1×/week.

### *M. tuberculosis* Genotyping

Available *M .tuberculosis* isolates were genotyped by spoligotyping (*19*,*20*), mycobacterial interspersed repetitive units (MIRUs) (*21*,*22*), and IS*6110* restriction fragment length polymorphism (RFLP) analysis (*23*). A cluster was defined as >2 *M. tuberculosis* isolates that had identical spoligotyping and MIRU results and for which RFLP results were identical or differed by only 1 band. To assess whether the proportion of MDR TB isolates that clustered was greater than that among pansusceptible isolates, we used the Fisher exact test with a significance level of 0.05.

### Mapping Patient Households

To assess geographic clustering of cases, we used global positioning system (GPS) technology to map TB patient households in the camp according to drug-susceptibility testing results. GPS data were analyzed with a spatial scan statistic that uses a varying-sized cylinder to encapsulate cases within the radius of the cylinders and calculates a p value and log likelihood ratio to determine the statistical significance of any clusters that may be detected (*24*).

### Tuberculin Skin Testing

Refugees received tuberculin skin tests (TSTs) to evaluate latent TB infection. Induration >5 mm was considered a positive test result (*25*). To assess recent transmission in the camp, we summarized and compared TST results for 3 categories of contacts: 1) a housemate or family member of a TB patient with at least 1 sputum smear-positive result for acid-fast bacilli; 2) a housemate or family member of a TB patient with only sputum smear-negative results; or 3) not a housemate or family member of a TB patient. Using those who were not a housemate or family member of a TB patient as the referent group, we calculated prevalence ratios and 95% confidence intervals (CIs) for each exposure group.

## Results

From April 2004 through January 2005, TB was diagnosed for 272 of the 15,455 refugees screened (Table 1). All 272 persons with a TB diagnosis were tested for HIV infection; only 1 was infected. Sputum-smear acid-fast bacilli results were available for 247 TB patients; 34 (13.8%) were positive. Culture results were available for 242 TB patients; 57 (23.6%) patients had positive culture results for *M. tuberculosis.*

**Table 1:** Demographic and disease characteristics among Hmong refugees with tuberculosis, Thailand, February 2005*

Characteristic	No. (%)
Total	272 (100)
Sex	
F	112 (41.2)
M	160 (58.8)
Age, y	
<15	21 (7.7)
15–64	153 (56.3)
>65	98 (36.0)
Culture results	
Positive	57 (21.0)
Negative	185 (68.0)
Unknown	30 (11.0)
DST results	
MDR TB	24 (8.8)
Other patterns	9 (3.3)
Pansusceptible	24 (8.8)
Smear results	
Ever positive	34 (12.5)
Always negative	213 (78.3)
Unknown	25 (9.2)

*DST, *Mycobacterium tuberculosis* drug-susceptibility test results; MDR TB, multidrug-resistant tuberculosis.

Drug-susceptibility testing found that 24 (42.1%) isolates were pansusceptible, 24 (42.1%) were MDR TB, and 9 (15.8%) were resistant to >1 anti-TB medications but were not MDR TB. Drug resistance was found in 9 patterns, 4 of which were MDR TB. Several additional resistance patterns were noted among the MDR TB isolates: streptomycin (n = 4); streptomycin and ethambutol (n = 15); streptomycin and pyrazinamide (n = 2); streptomycin, ethambutol, and pyrazinamide (n = 3). Of the 24 MDR TB patients, 15 (62.5%) had positive sputum smear results.

### Patient Interviews

Of 272 TB patients, 241 (88.6%) were interviewed. Treatment for TB before pre-immigration screenings began in April 2004 was reported by 15 (6.2%); none had received directly observed therapy (DOT). Of the 15 previously treated patients, 3 (20.0%) had a current diagnosis of MDR TB, 1 (6.7%) had TB with isoniazid resistance only, 1 (7.0%) had TB that was pansusceptible, and 10 (66.7%) had received clinical diagnoses without culture confirmation. All 3 MDR TB patients who had a history of previous treatment had received their treatment during the 3 years before their current diagnosis.

After pre-immigration TB screenings started, some camp residents visited healthcare providers outside the camp to seek treatment for conditions that might have precluded their passing the pre-immigration medical examination. Chest radiograph screenings were obtained outside the camp by 39 (16.2%) patients during the period between when medical screening began at the camp and when their own pre-immigration screening was scheduled. Of these, 32 (82.1%) took TB medications during this time; only 9 (28.1%) reported that they were told by a doctor or nurse that they had TB. Among the remaining 23 patients not reporting a diagnosis of TB, 1 (4.3%) had MDR TB, 1 (4.3%) had streptomycin-resistant TB, 2 (8.7%) had pansusceptible TB, and the remaining 19 (82.6%) were diagnosed clinically without culture confirmation.

**Figure 1:**
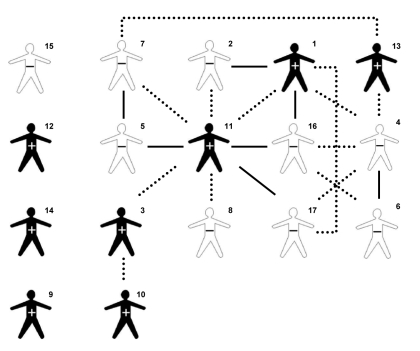
Social links between Hmong refugees with multidrug-resistant tuberculosis, Thailand, February 2005. Numerals indicate patients, in order of diagnosis. +, smear positive; –, smear negative; dotted lines, weak link; solid lines, strong link.

Of the 24 MDR TB patients, 17 (70.8%) responded to the questions regarding social links to other MDR TB patients (Figure 1.) Among these, 9 (52.9%) reported having at least 1 strong link with another MDR TB patient, and 4 MDR TB patients (23.5%) reported having at least 2 strong links with another MDR TB patient. One patient who had sputum smear-positive TB (Figure 1, patient 11) was central to a social network that linked 13 (76.5%) patients. The 3 MDR TB patients that had been previously treated for TB (Figure 1, patients 1, 2, and 3,) were all directly linked to patient 11 and included in the 13-patient network.

### TB Genotyping

Genotyping results were available for 46 (80.7%) of the 57 culture-confirmed cases. Of these, 30 (65.2%) belonged to 1 of 5 clusters (clusters A–E), which ranged from 2 to 20 matching isolates. The remaining 16 (34.8%) isolates were unique (Table 2). The largest culture, cluster C, had 20 cases, all of which were MDR TB; they represented 87.0% of the 23 MDR TB isolates with genotyping results. The cluster C spoligotype pattern was 000000000003771, and the MIRU pattern was 223325173533. Isolates in cluster C had a 21-band RFLP result. Of the MDR TB isolates that were not included in cluster C, 2 had spoligotype and RFLP results that matched those included in cluster C, but according to MIRU results, they differed at 1 locus. The third MDR TB isolate not included in cluster C differed according to both MIRU and RFLP results. MDR TB isolates were more likely than non–MDR TB isolates to cluster (Fisher exact p = 0.02).

**Table 2:** *Mycobacterium tuberculosis* drug-susceptibility test results, by genotype, Hmong refugee tuberculosis patients, Thailand, February 2005*

Genotype cluster	Isolate results			
	Some resistance	MDR TB	Pansusceptible	Total
Unique isolates	5	3	8	16
A	0	0	3	3
B	0	0	2	2
C	0	20	0	20
D	0	0	2	2
E	1	0	2	3
Total	6	23	17	46

*MDR TB, multidrug-resistant tuberculosis.

Among the 17 MDR TB patients who responded to the questions regarding social links to other MDR TB patients (Figure 1), 15 (88.2%) were included in cluster C. Isolates from patients 5 and 12 matched cluster C according to spoligotype and RFLP results but differed at 1 locus according to MIRU results.

As of 2007, genotyping results were available for 7 additional patients from this camp who had received a diagnosis of MDR TB after arrival in the United States (California). According to spoligotyping and MIRU results, isolates collected from 6 of these patients matched the cluster C strain. The seventh isolate had the same spoligotype result but differed at 1 locus according to MIRU.

### Patient Household Maps

**Figure 2:**
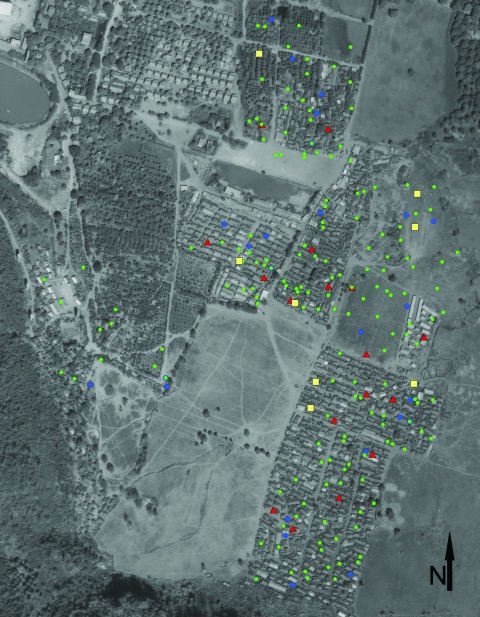
Locations of dwellings within camp for Hmong refugees with tuberculosis (TB), Thailand, February 2005. Symbols indicate dwellings of patients with the following types of TB: red triangles, multidrug-resistant; yellow squares, resistant to >1 anti-TB medications but not MDR TB; blue circles, pansusceptible; green circles, unknown drug-susceptibility testing results.

TB patients were widely distributed throughout the camp living quarters (≈0.5 km^2^) (Figure 2). The spatial analysis showed 3 nonsignificant spatial clusters (p>0.05), which suggests no significant geographic clustering of TB patient households, either among all patients or among subsets with similar drug-susceptibility testing patterns.

### TST Results

Of the 5,637 camp residents that had TSTs performed and results read, 1,624 (28.8%) had positive results. Among those who were family members or housemates of a camp patient with a sputum smear-positive TB diagnosis, 96 (44.0%) had positive TST results. These contacts were 1.6× (95% CI 1.4–1.9) as likely as the referent group to have a positive result. There was an increased risk (not statistically significant) for a positive result associated with being a family member or housemate of a patient with sputum smear–negative disease (relative risk = 1.1, 95% CI 1.0–1.2). After controlling for patients’ sputum smear status, we found that household contacts of patients with MDR TB, pansusceptible TB, and TB with some drug resistance had the same risk for a positive TST result.

### Modifications and Enhancements to Pre-immigration TB Screening and Treatment

After the investigation in February 2005, recommendations for pre-immigration TB screening and treatment for Hmong refugees in Thailand were again modified and enhanced (Table 3). These modifications required that all refugees >6 months of age be screened with chest radiography. Persons with suspected TB had 3 sputum specimens collected for smear microscopy, culture, and drug-susceptibility testing with rapid, liquid-based methods. All patients were required to show documentation of having received DOT for the duration of their TB treatment before they were permitted to travel to the United States. In addition, a TB culture laboratory was built within the camp, mechanisms were developed to import quality-assured second-line TB medications, and nurses and physicians in the camp received advanced training in TB diagnosis and treatment. Finally, although TSTs were performed for all camp residents during this investigation, the enhanced TB screening algorithm required that only those 6 months to 10 years of age receive TST.

**Table 3:** Summary of tuberculosis screening algorithm components for Hmong refugees, by date, Thailand*

Date	Medical history	Physical examination	TST			Sputum smear	Culture	Drug-susceptibility testing
				CXR				
				1 view	2 views			
Apr–Jun 2004	Yes	Yes	No	Yes, for those >15 years of age	No	Yes, if CXR indicated possible TB	Yes, if sputum-smear positive for AFB	No
Jul 2004–Jan 2005	Yes	Yes	No	Yes, for those >15 years of age	No	Yes, if CXR indicated possible TB	Yes, regardless of sputum-smear status	Yes
Feb 2005–Apr 2007	Yes	Yes	Yes, for those 6 mo to 10 y of age	Yes, for those >10 years of age	Yes, for those 6 mo to 10 y of age	Yes, if CXR indicated possible TB	Yes, regardless of sputum-smear status	Yes

*TST, tuberculin skin test; CXR, chest radiograph; TB, tuberculosis; AFB, acid-fast bacilli.

After implementation of the final enhanced TB screening and treatment requirements, 97 additional TB cases, including 2 MDR TB, were diagnosed in Thailand, resulting in an overall total of 369 TB cases. As of April 2007, health departments in the United States reported 46 cases of TB, 6 of which were MDR TB, among 9,455 Hmong refugees who immigrated to the United States before implementation of enhanced screening (487 cases/100,000 persons). In contrast, 4 cases of TB, 1 of which was MDR TB, have been diagnosed in the United States among the 5,705 Hmong refugees who immigrated after implementation of enhanced screening (70 cases/100,000 persons). The proportion of cases diagnosed in the United States after the enhanced screening was significantly lower than the proportion diagnosed before the enhanced screening (Fisher exact test p<0.001).

## Discussion

An outbreak of MDR TB occurred among a population in which TB rates were already elevated; as a result, TB (some MDR TB) was imported into the United States. Several lines of evidence support the conclusion that this was an outbreak. First, 13 (76.5%) of 17 MDR TB patients interviewed reported having had recent and regular exposure to another MDR TB patient. Although GPS did not demonstrate geographic clustering, lack of clustering is not unexpected because the camp was small and its population density was high, making social networks, rather than absolute physical distance between dwellings, the most important facilitator of TB transmission. Second, 20 (87.0%) of 23 MDR TB isolates were strains that matched by 3 different molecular subtyping methods. It is possible that 2 additional isolates (22 total) were part of the outbreak as well because they matched the outbreak strain according to spoligotyping and RFLP results and differed by only 1 locus according to MIRU; therefore, this difference may represent a change in genotype in the same clone over time (*26*). Third, elevated rates of tuberculin reactivity in household contacts of smear-positive patients suggest substantial recent transmission, not simply endemic disease. As rates of tuberculin reactivity were elevated in household contacts of all smear-positive patients, transmission of pansusceptible TB, TB with drug-resistance but not MDR TB, and MDR TB likely occurred in the camp before and during the initial resettlement of refugees to the United States in 2004. Rates of TST positivity were not presumed to be inflated by vaccination with *M. bovis* BCG, because there was no indication or documentation of BCG vaccination among this group of refugees.

Delayed recognition of TB transmission in the camp had several negative consequences: increased number and severity of cases among refugees and importation of TB, including MDR TB, into the United States. In addition, the costs incurred by the US government were substantial and resulted from halting immigration (e.g., cancellation of flights, emergency overseas investigations, and program enhancements), public health investigations in the United States and Thailand, and medical costs of diagnosis and treatment. Because half of the cases of MDR TB were sputum smear–negative for acid-fast bacilli, had sputum smear microscopy continued to be used as the only tool for initial screening, the outbreak of MDR TB would likely have gone undetected and many more MDR TB cases would have been imported into the United States.

Hmong refugees who had a positive TST result did not receive treatment for latent TB infection before immigrating to the United States because they were not contagious and thus were eligible to travel on commercial airplanes. No universally accepted standard therapy is available for latent TB infection with a suspected MDR TB strain. Rather, therapy for suspected latent infection from MDR TB is determined on a case-by-case basis in accordance with drug-susceptibility testing results from the putative source. After arrival in the United States, the receiving local health department jurisdictions decided how to evaluate, reevaluate, and treat patients with latent TB infection. Those who had no known contact with an MDR TB patient were treated with isoniazid; those who had had contact with an MDR TB patient, either overseas or in California, were treated with an MDR TB contact treatment regimen tailored to the source case isolate’s susceptibility pattern. Most often, they were treated with fluoroquinolone and pyrazinamide because TB isolates from Hmong refugees were resistant to isoniazid, rifampin, and ethambutol. If a patient refused medication for latent TB infection, that patient was closely clinically monitored for 2 years.

This outbreak led to major changes in public health practice for this refugee group and in future pre-immigration medical screening policies. Enhancements to pre-immigration TB screening (Table 3) contributed to a reduction in the number of imported TB cases. As a result, CDC is working with the US Department of State, panel physicians, the International Organization for Migration, and other organizations to implement similar enhancements to general pre-immigration TB screening guidelines. These new technical instructions are being implemented first in priority countries, as determined by immigration patterns and TB prevalence. Eventually, all refugees and immigrants entering the United States will be screened with a revised TB screening algorithm that includes mycobacterial culture and susceptibility testing. Since the end of 2007, applicants for US immigration who have been screened according to the new technical instructions have originated from Mexico, the Philippines, Nepal, and Thailand. CDC notifies US state and local health departments when panel physicians in a specific country begin implementing this revised algorithm. Ongoing US national TB surveillance will help determine the effect of this effort on reducing the number of foreign-born persons with TB living in the United States. In this outbreak investigation, 3 MDR TB patients were identified who had received treatment in the past 3 years at healthcare facilities outside the refugee camp; however, none reported having received DOT, the strategy recommended for reducing emergence of drug resistance (*27*). Our findings support the goals of the World Health Organization’s second Global Plan to Stop TB, which includes refugees as a high-risk group requiring attention by TB control programs. This outbreak also highlights the need for US public health preparedness efforts to focus on containment of threats of emergent diseases, such as MDR TB, at their source (*28*).

To control TB and prevent MDR TB, multiple organizations—including government agencies, multilateral agencies, and nongovernment organizations—must work together to provide high-quality TB diagnosis and treatment consistent with international standards of care in both host and receiving countries (*29*). For low-incidence countries, such as the United States, investing in global TB control is a cost-effective strategy for reducing TB, domestically and globally (*30*,*31*).
